# Acute kidney injury in nonagenarians: clinical characteristics and mortality

**DOI:** 10.1590/2175-8239-JBN-2023-0088en

**Published:** 2024-05-24

**Authors:** Rafael Peixoto Lima Dias, Daniella Bezerra Duarte, Danilo de Castro Bulhões Mascarenhas Barbosa, Rodrigo Peixoto Campos

**Affiliations:** 1 Centro Universitário Tiradentes, Faculdade de Medicina, Maceió, AL, Brazil.; 2 Universidade Federal de Alagoas, Faculdade de Medicina, Maceió, AL, Brazil.; 3 Santa Casa de Misericórdia de Maceió, Instituto de Nefrologia Ribamar Vaz, Maceió, AL, Brazil.

**Keywords:** Nonagenarians, Acute Kidney Injury, Mortality, Nonagenários, Injúria Renal Aguda, Mortalidade

## Abstract

**Introduction:**

Nonagenarians constitute a rising percentage of inpatients, with acute kidney injury (AKI) being frequent in this population. Thus, it is important to analyze the clinical characteristics of this demographic and their impact on mortality.

**Methods:**

Retrospective study of nonagenarian patients with AKI at a tertiary hospital between 2013 and 2022. Only the latest hospital admission was considered, and patients with incomplete data were excluded. A logistic regression analysis was conducted to define risk factors for mortality. A p-value < 0.05 was considered statistically significant.

**Results:**

A total of 150 patients were included, with a median age of 93.0 years (91.2–95.0), and males accounting for 42.7% of the sample. Sepsis was the most common cause of AKI (53.3%), followed by dehydration/hypovolemia (17.7%), and heart failure (17.7%). ICU admission occurred in 39.3% of patients, mechanical ventilation in 14.7%, vasopressors use in 22.7% and renal replacement therapy (RRT) in 6.7%. Death occurred in 56.7% of patients. Dehydration/hypovolemia as an etiology of AKI was associated with a lower risk of mortality (OR 0.18; 95% CI 0.04–0.77, p = 0.020). KDIGO stage 3 (OR 3.15; 95% CI 1.17–8.47, p = 0.023), ICU admission (OR 12.27; 95% CI 3.03–49.74, p < 0.001), and oliguria (OR 5.77; 95% CI 1.98–16.85, p = 0.001) were associated with mortality.

**Conclusion:**

AKI nonagenarians had a high mortality rate, with AKI KDIGO stage 3, oliguria, and ICU admission being associated with death.

## Resumo

**Introdução:**

Nonagenários constituem um percentual de pacientes internados em ascensão, sendo a injúria renal aguda (IRA) frequente nesses pacientes. Sendo assim, é importante analisar as características clínicas dessa população e seu impacto na mortalidade.

**Métodos:**

Estudo retrospectivo de pacientes nonagenários com IRA entre 2013 e 2022 em um hospital terciário. Apenas o último internamento foi considerado e pacientes com dados incompletos foram excluídos. Uma análise por regressão logística foi realizada para definir fatores de risco para mortalidade. Um valor de p < 0,05 foi considerado significativo.

**Resultados:**

Foram incluídos 150 pacientes com mediana de idade 93,0 anos (91,2–95,0) e sexo masculino em 42,7%. Sepse foi a causa mais comum de IRA (53,3%), seguida de desidratação/hipovolemia (17,7%) e insuficiência cardíaca (17,7%). Admissão na UTI ocorreu em 39,3% dos pacientes, ventilação mecânica em 14,7%, uso de vasopressores em 22,7% e realização de terapia renal substitutiva (TRS) em 6,7%. Óbito ocorreu em 56,7% dos pacientes. Desidratação/hipovolemia como etiologia da IRA foi associado a menor risco de mortalidade (OR 0,18; IC 95% 0,04–0,77, p = 0,020). Estágio KDIGO 3 (OR 3,15; IC 95% 1,17–8,47, p = 0,023), admissão na UTI (OR 12,27; IC 95% 3,03–49,74, p < 0,001) e oligúria (OR 5,77; IC 95% 1,98–16,85, p = 0,001) foram associados à mortalidade.

**Conclusão:**

Nonagenários com IRA apresentaram alta mortalidade e IRA KDIGO 3, oligúria e admissão na UTI foram associadas ao óbito.

## Introduction

In recent decades, the increase in life expectancy and aging of the population have raised the number of elderly people admitted to hospitals^1^. Among the elderly, the population aged over 80 is the fastest-growing demographic^2^. Nevertheless, there are still few studies on patients aged over 90 years, and the available data are inconclusive regarding the profile of comorbidities and prognosis in this population^3^.

With aging, different physiological and functional changes occur in the kidneys, such as a reduction in glomerular filtration rate, loss of cortical volume and nephrosclerosis^4^. Advanced age and polypharmacy may be considered risk factors for kidney diseases, and in hospitalized patients, Acute Kidney Injury (AKI) is a prominent condition in the elderly, altering the individual’s prognosis^3,4,5^.

Among the elderly, nonagenarians seem to have unique characteristics, comorbidities and clinical outcomes^2,3,5,6^. The incidence of AKI is high in patients in this age group hospitalized for any underlying pathology^3^. However, published studies on AKI in nonagenarians are still insufficient. A recent study conducted in Brazil, including 436 nonagenarians admitted to hospital, identified an AKI incidence of 45%. The mortality rate was 66.8% in patients with AKI, and only 23.8% among those who did not develop AKI (p < 0.001)^6^. The clinical profile of nonagenarian patients who develop AKI is poorly understood, and more data on the subject is needed in order to broaden the scientific basis available to medical professionals.

The aim of this study is to analyze the clinical characteristics and risk factors associated with mortality in a population of nonagenarian patients with AKI admitted to a tertiary hospital.

## Method

### Study Design and Patient Selection

Retrospective study of patients who developed AKI between January 2013 and December 2022 at a tertiary hospital (Instituto de Nefrologia Ribamar Vaz – Santa Casa de Misericórdia de Maceió, Maceió, Alagoas, Brazil). All patients assessed by the nephrology team following a consultation referral who presented with AKI during this period were included in a database, totaling 1528 patients. Only patients aged 90 years or older were initially included, with a total of 159 patients. Kidney transplant recipients and individuals with incomplete clinical data were excluded. Only the latest hospital admission was considered. Thus, 150 patients were included for final analysis. All clinical information was collected through electronic medical records. The study was approved by the local research ethics committee (CAAE 67867222.5.0000.5641).

### Definitions

AKI was defined using the Kidney Disease Improving Global Outcomes (KDIGO) diagnostic criteria and classification^7^. AKI is defined as an increase ≥0.3 mg/dL in baseline serum creatinine within 48 h, a 1.5 to 1.9-fold increase in baseline serum creatinine (if known or assumed to be that of the last seven days) or a reduction in urine output <0.5 mL/Kg for at least 6 h (KDIGO 1). KDIGO stage 2 is defined as an increase in serum creatinine of 2 to 3 times the baseline value or a reduction in urine output <0.5 mL/Kg for ≥12 h. KDIGO stage 3 is considered if there is an increase in creatinine greater than or equal to 3 times the baseline value, or an increase ≥4 mg/dL, or initiation of RRT, or urine output <0.3 mL/Kg for ≥24 h, or anuria for ≥12 hours. The etiology was defined by the nephrologist who evaluated and registered the patient in the database. If multifactorial AKI was suspected, the nephrologist would determine the main cause and register it. The diagnosis of chronic kidney disease was also defined based on clinical and laboratory data from medical records and evolution during hospitalization. Previous glomerular filtration rate lower than 60 mL/min/1.73m^2^ or albuminuria greater than 30 mg/g was used to define CKD, and patients were classified as having AKI superimposed on CKD. KDIGO guidelines were used to classify the stage of CKD. The renal replacement therapy (RRT) method was indicated by the nephrologist who evaluated and recommended treatment, either intermittent hemodialysis, prolonged hemodialysis or continuous therapy (hemodiafiltration). Oliguria was defined as diuresis <400 mL in 24 h. The Charlson Comorbidity Index assesses an individual’s 10-year survival based on their age and the presence of 17 comorbidities^8^.

### Statistical Analysis

Statistical analysis was performed using IBM SPSS^®^ version 20.0. Data were presented as mean ± standard deviation, median (1st and 3rd quartile), and percentage rates. Comparison between variables normally distributed was performed using the Student’s t-test, and variables with non-normal distribution were compared using the Mann-Whitney U test. The chi-square test was used to analyze categorical variables. To analyze whether certain variables could affect death in this population, univariate and multivariate analyses were performed using logistic regression. All variables with p < 0.10 in the univariate analysis were included in the multivariate analysis model. Logistic regression data were presented as odds ratios (OR) and 95% confidence intervals (95% CI). A p-value was considered significant when <0.05.

### Results

A total of 150 patients were included in the study. They had a median age of 93.0 years (91.2–95.0). According to KDIGO stages, patients were classified as follows: KDIGO 1, 56 patients (37.3%); KDIGO 2, 29 patients (19.3%); and KDIGO 3, 65 patients (43.3%). Of the total number of patients, 60 (40%) had CKD. ICU admission occurred in 59 patients (39.3%), mechanical ventilation in 22 (14.7%), use of vasopressors in 34 (22.7%), and RRT in 10 (6.7%). The most common AKI etiology was sepsis in 80 patients (53.3%), followed by dehydration/hypovolemia (17.3%), heart failure (17.3%), and urinary obstruction (5.3%). Table 1 shows the clinical and demographic characteristics of the study group.

**Table 1.** Clinical and demographic characteristics of a 150 nonagenarian sample

Variables	n.	%
Male	64	42.7
Age (years)		
90–93	90	60.0
94–96	37	24.7
≥97	23	15.3
CKD	60	40.0
2	7	4.7
3a	6	4.0
3b	21	14.0
4	21	14.0
5	5	3.3
Length of stay (weeks)		
<1	37	24.7
1–2	43	28.7
2–4	42	28.0
>4	28	18.7
Etiology		
Sepsis	80	53.3
Dehydration/hypovolemia	26	17.3
Heart failure	26	17.3
Urinary obstruction	8	5.3
Nephrotoxicity	4	2.7
Covid-19	4	2.7
Hepatorenal	1	0.7
Glomerulonephritis	1	0.7
KDIGO stage		
KDIGO 1	56	37.3
KDIGO 2	29	19.3
KDIGO 3	65	43.3
Charlson Comorbidity Index		
≥6	70	46.7
≥7	26	17.3
ICU admission	59	39.3
Mechanical ventilation	22	14.7
Vasopressor use	34	22.7
Oliguria	59	39.3
Renal Replacement Therapy	10	6.7
Death	85	56.7

Legend: CKD – chronic kidney disease; ICU – intensive care unit; KDIGO – Kidney Disease Improving Global Outcomes.

In-hospital death occurred in 85 patients (56.7%). The following characteristics were more frequently observed in the mortality group: sepsis (61.2% vs 43.1%, p = 0.028), KDIGO 3 (56.5% vs 26.2%, p < 0.001), ICU admission (61.2% vs 10.8%, p < 0.001), mechanical ventilation (22.4% vs 4.6%, p = 0.002), vasopressor use (34.1% vs 7.7%, p < 0.001), oliguria (60% vs 12.3%, p < 0.001), and RRT (10.6% vs 1.5%, p < 0.028). CKD (31.8% vs 50.8%, p = 0.019), dehydration/hypovolemia (7.1% vs 30.8%, p < 0.001), and KDIGO 1 (28.1% vs 49.2%, p = 0.008) were more common in the non-mortality group (Table 2).

**Table 2.** Stratified mortality according to clinical and demographic variables

Variables	Mortality		p-value
	Yes n = 85	No n = 65	
Male n. (%)	33 (38.5)	31 (47.7)	0.276
Age (years)*	92.7 (91.0-95.0)	93.4 (91.6-95.0)	0.341
≥94 years n. (%)	32 (37.6)	28 (43.1)	0.501
≥97 years n. (%)	13 (15.3)	10 (15.4)	0.988
CKD n. (%)	27 (31.8)	33 (50.8)	**0.019**
Length of stay (days)*	15.0 (8.0-24.0)	12 (7.0-25.0)	0.483
>2 weeks n. (%)	46 (54.1)	28 (43.1)	0.180
Etiology n. (%)			
Sepsis	52 (61.2)	28 (43.1)	**0.028**
Dehydration/hypovolemia	6 (7.1)	20 (30.8)	**<0.001**
Heart failure	15 (17.6)	11 (16.9)	0.908
KDIGO stage n. (%)			
KDIGO 1	24 (28.2)	32 (49.2)	**0.008**
KDIGO 2	13 (15.3)	16 (24.6)	0.152
KDIGO 3	48 (56.5)	17 (26.2)	**<0.001**
Charlson*	5.0 (5.0-6.0)	5.0 (5.0-6.0)	0.886
≥7 n. (%)	19 (22.4)	7 (10.8)	0.063
ICU admission n. (%)	52 (61.2)	7 (10.8)	**<0.001**
Mechanical ventilation n. (%)	19 (22.4)	3 (4.6)	**0.002**
Vasopressor use n. (%)	29 (34.1)	5 (7.7)	**<0.001**
Oliguria n. (%)	51 (60.0)	8 (12.3)	**<0.001**
Renal Replacement Therapy n. (%)	9 (10.6)	1 (1.5)	**0.028**

Legend: CKD – chronic kidney disease; ICU – intensive care unit; KDIGO – Kidney Disease Improving Global Outcomes. *Median (1st and 3rd quartile).

In the univariate logistic regression analysis, the following variables were associated with mortality: sepsis (OR 2.08, 95% CI 1.08–4.02; p = 0.029), KDIGO 3 (OR 3.76, 95% CI 1.75–8.10; p < 0.001), ICU admission (OR 13.05, 95% CI 5.32–32.03; p < 0.001), mechanical ventilation (OR 5.95, 95% CI 1.68–21.10; p = 0.006), vasopressor use (OR 6.21, 95% CI 2.25–17.17; p < 0.001), and oliguria (OR 10.69, 95% CI 4.53–25.20; p < 0.001). Presence of CKD (OR 0.45, 95% CI 0.23–0.88; p = 0.019) and dehydration/hypovolemia (OR 0.17, 95% CI 0.06–0.46; p < 0.001) were associated with a lower risk of death in the univariate analysis. In multivariate analysis, only dehydration/hypovolemia as an etiology of AKI remained unassociated with the risk of mortality (OR 0.18, 95% CI 0.04–0.77; p = 0.020). However, KDIGO stage 3 (OR 3.15, 95% CI 1.17–8.47; p = 0.023), ICU admission (OR 12.27, 95% CI 3.03–49.74; p < 0.001), and oliguria (OR 5.77, 95% CI 1.98–16.85; p = 0.001) remained associated with mortality. RRT was not associated with the risk of death in either the univariate or multivariate analysis (Table 3).

**Table 3.** Mortality predictors by univariate and multivariate logistic regression

Variables	Univariate	p-value	Multivariate	p-value
	OR (95% CI)		OR (95% CI)	
Male	0.70 (0.36–1.34)	0.277	–	–
Age ≥ 94 years	0.80 (0.41–1.54)	0.501	–	–
CKD	0.45 (0.23–0.88)	0.019	0.82 (0.33–2.04)	0.669
Hospital stay > 2 weeks	0.64 (0.33–1.23)	0.181	–	–
Etiology				
Sepsis	2.08 (1.08–4.02)	**0.029**	0.62 (0.22–1.75)	0.372
Dehydration/hypovolemia	0.17 (0.06–0.46)	**<0.001**	0.18 (0.04–0.77)	**0.020**
Heart failure	1.05 (0.45–2.37)	0.908	–	–
KDIGO stage				
KDIGO 1 (reference)	1	–	–	–
KDIGO 2	1.08 (0.44–2.67)	0.862	–	–
KDIGO 3	3.76 (1.75–8.10)	**<0.001**	3.15 (1.17–8.47)	**0.023**
Charlson ≥ 7	2.38 (0.94–6.08)	0.069	1.60 (0.50–5.17)	0.431
ICU admission	13.05 (5.32–32.03)	**<0.001**	12.27 (3.03–49.74)	**<0.001**
Mechanical ventilation	5.95 (1.68–21.10)	**0.006**	0.56 (0.08–4.18)	0.562
Vasopressor use	6.21 (2.25–17.17)	**<0.001**	0.86 (0.14–5.40)	0.864
Oliguria	10.69 (4.53–25.20)	**<0.001**	5.77 (1.98–16.85)	**0.001**
Renal Replacement Therapy	7.58 (0.94–61.42)	0.058	0.52 (0.04–6.11)	0.601

Legend: CKD – chronic kidney disease; OR – Odds ratio; CI – confidence interval; ICU – intensive care unit; KDIGO – Kidney Disease Improving Global Outcomes. Variables included in the multivariate logistic regression: CKD, sepsis, dehydration/hypovolemia, KDIGO 3, Charlson ≥ 7, ICU admission, mechanical ventilation, vasopressor use, oliguria, renal replacement therapy.

It is important to highlight that although only 10 patients underwent RRT treatment, another 45 patients were discussed for the possibility of initiating therapy. However, RRT was contraindicated because it was considered a futile treatment in 40 patients (88.9%) and due to patient or family refusal in 5 patients (11.1%). Mortality in this group occurred in 43 patients (95.3%).

## Discussion

Advancing age leads to several clinical and epidemiological differences between young and elderly patients. Even among the elderly, age groups should be considered in the patient’s clinical management, since studies have shown differences in clinical profile and outcomes when comparing different age groups among patients aged 60 or older^9,10,11^.

In the studied group, there was a high prevalence of CKD patients (40%). Published studies suggest that these patients have a higher incidence of AKI^12^. The primary AKI etiology was sepsis, similar to the over-60s population in other studies ^13,14^, followed by dehydration/hypovolemia and heart failure.

In-hospital mortality in nonagenarians with AKI according to published articles ranges from 23% to 70%, depending on mean age, patient severity, comorbidity profile and criteria for defining AKI^3,4,6^. In our study, we observed a mortality rate of 56.7%, similar to other published data, which may be justified by the prevalence of comorbidities and severity of the study group. Although 90% of patients who underwent RRT died, the treatment was not a predictor of mortality, possibly due to the small number of patients undergoing the procedure (10 patients). Higher scores on the Charlson Comorbidity Index were not related to mortality in our cohort, and despite the divergence from other data observed in a similar population^15^, the utility of this tool for predicting mortality in advanced age is questionable^16^.

In the surviving group, there was a high prevalence of CKD, KDIGO 1 and dehydration/hypovolemia. Data suggest that CKD patients who develop AKI have a lower risk of death^17^, and are not associated with worse outcomes^3^. In our study, although CKD was more prevalent in the survivor group (50.8%, p = 0.019), it was not identified as a lower risk factor for mortality in the multivariate analysis. Prolonged hospitalizations were not associated with higher mortality in our cohort. This result differs from that published in another Brazilian study that evaluated AKI in nonagenarians^6^.

Only KDIGO 3, ICU admission and oliguria remained as independent risk factors for mortality in multivariate analysis. An independent association with mortality has been demonstrated for all KDIGO stages, with a greater risk at higher stages^6^, as well as a reported relationship between nonagenarians with more severe AKI stages and mortality^3^. In our cohort, only KDIGO 3 had this association, with a three-fold higher chance of mortality for the individual. Admission of elderly people with AKI to the ICU was considered a risk factor for mortality in previously published data^6,18^. Our study demonstrated a twelve-fold increased risk for these cases. Some studies on the elderly have described an association of oliguria with an increased risk of death^18,19^. However, this factor has not been evaluated in studies on nonagenarians. In our analysis, this factor led to an almost six-fold increased risk for mortality. Dehydration/hypovolemia as a cause of AKI was associated with a lower risk of death.

Published studies report that the need for RRT in elderly people with AKI ranges from 3.5% to 39.5%. This variation may be related to the number of patients with advanced AKI stages in each study^20,21,22,23^. In our study, 55 (36.7%) patients had clinical and/or laboratory indications for RRT initiation prescribed by the medical team. However, in 45 (30%) patients RRT was contraindicated due to treatment futility or family refusal, and only 10 (6.7%) underwent treatment.

A publication that evaluated the outcome of 703 AKI patients, with a mean age of 63.5 ± 14.8 years, and requiring dialysis, reported a mortality rate of 42%^24^. Our study showed a 90% mortality rate in those who underwent RRT, similar to another study that evaluated patients over 90 years of age^6^. RRT was not an independent risk factor for mortality possibly because only 10 patients underwent treatment; thus, we cannot extrapolate the impact of treatment on mortality. The use of RRT in elderly patients, especially those over 90, is a medical practice dilemma, as studies are still searching for precise indication criteria for a survival outcome in those undergoing such an invasive procedure^25,26,27^.

This study has some limitations. It was a single-center study, limiting data extrapolation to other centers; it had a retrospective design, restricting the collection of data available in electronic medical records and leading to the exclusion of patients with incomplete data; and also because we only selected the last admission of patients, which may indicate greater severity in this last hospitalization.

## Conclusion

The presence of hospitalized nonagenarians is a reality nowadays, and AKI has a high incidence in this group, in addition to being associated with a high mortality rate. In this retrospective analysis, AKI KDIGO stage 3, oliguria, and ICU admission were risk factors associated with death. AKI due to dehydration/hypovolemia was associated with a lower risk of death. Further studies are needed in nonagenarians with AKI to better understand clinical needs and define optimal treatment.
